# The Clinical and Medicolegal Analysis of Electrical Shocked Rats: Based on the Serological and Histological Methods

**DOI:** 10.1155/2016/4896319

**Published:** 2016-08-25

**Authors:** Huitong Liu, Qiaofeng Wang, Ze Zhao, Yanan Xie, Suzhen Ding, Zhenyuan Wang

**Affiliations:** ^1^Shaanxi Provincial People's Hospital, The Third Affiliated Hospital of Xi'an Jiaotong University, Xi'an, China; ^2^School of Forensic Medicine, Xi'an Jiaotong University Health Science Center, Xi'an, China; ^3^Department of Neurology, Huashan Hospital, Fudan University, Shanghai, China

## Abstract

This research was aimed at discovering the serological and histological changes in cardiac and hepatic tissue after electric shock. The CK-MB, ALT, and AMS indexes were tested with serological methods. Moreover, the Bcl-2, Bax, and Hsp-60 expression levels were carefully measured. An electrical injury model was established by giving rats electric shocks at 110 V with alternating electric current. Blood samples from the rats were analyzed for the biochemical indexes. The degrees of pathological changes in the heart and liver were evaluated using IHC staining for Bcl-2, Bax, and Hsp-60. The levels of CK-MB in the electrical injury group rapidly peaked at 0.5 hours after the electric shock. Additionally, the levels of Bcl-2, Bax, and Hsp-60 in the cardiac and hepatic tissues changed regularly after the electrical injury and exhibited apparent differences from the levels in the control group. CK-MB, ALT, and AMS were altered regularly after electric shock, and these results provide significant information for clinical and medicolegal practice. This research has shed light on the assessment of electrical injury without obvious electrical burns. Furthermore, the findings obtained for Bcl-2/Bax and Hsp-60 can also facilitate pathological diagnosis and the identification of antemortem and postmortem electrical injury.

## 1. Introduction

Injury caused by electrical current, known as electrical injury, can occur easily and frequently due to the widespread use of electrical appliances [1, 2]. As a relatively infrequent phenomenon, electrical injury continues to present problems in clinical and medicolegal practice [3, 4]. Data revealed that 0.5% of deaths are related to electrical shock, and among these deaths, 60%–70% are caused by low voltage power supplies and occasionally the short circuiting of storage batteries in cars in America and China [5–8]. When an injury is accompanied by typical electrical burns, the diagnosis and conclusion are easy to make, but in cases with multiple other factors, such as interference from the environment, typical electrical burns may not exist. Consequently, the inability to diagnose whether the injuries were caused by electricity may also impede the treatment of the patients or investigations of deaths [1–6].

An easy method to detect electrical injury is necessary. Many studies have proved that electric shocks cause multisystem injuries and produce secondary regular variations at the cellular and molecular levels. Detecting the molecules in some particular organs and some enzymes may represent alternative methods to help diagnose and evaluate electrical injuries. These enzymes, including CK-MB, ALT, and AMS, provide an indirect index of injuries or illnesses in organs. Similarly, Bc1-2/Bax and Hsp-60 have been found to be related to cellular injury and repair. As the product of the Bc1-2 gene, Bc1-2/Bax can reflect the condition of living cells. Hsp-60 is an important member of the Hsp family, which is a group of highly conserved proteins that function in the dislocating, folding, and assembling of proteins as molecular chaperones.

In this work, we studied the changes in Bc1-2/Bax and Hsp by pathological detection and examined creatine kinase-MB (CK-MB), alanine transaminase (ALT), and amylase (AMS) by using serological methods to evaluate the degree of injury after electric shock. We also proved the existence of organ injury after electrical injury and the regularity of repairs. Additionally, we intended to find a plausible explanation for the postmortem differences between electrocution and electrical shock injury. We believe that these findings can contribute to both clinical diagnoses and forensic judgments in legal medicine.

## 2. Materials and Methods

### 2.1. Laboratory Animals and Grouping

A total of 228 male Sprague-Dawley rats (SPF, Certificate of Quality number 2008-008) purchased from the Animal Center of Xi'an Jiaotong University and weighing 160–200 g (average 180 g) were randomly divided into 2 groups, that is, (1) animals for the serological examinations and (2) animals for the pathological examinations. Because the serological examinations required 4–6 mL of blood, the removal of which caused organ ischemia in the rats that would have influenced the pathological examination, we divided the rats into two examination groups (Table 1).

The animals for the serological examinations were divided into 12 subgroups with 6 rats in each group: 0 hr, 0.25 hr, 0.5 hr, 1 hr, 2 hr, 3 hr, 4 hr, 5 hr, 6 hr, 7 hr, and 8 hr after electric shock subgroups and a blank subgroup without electric shock. The animals used for the pathological examinations were divided into 4 groups: an electrocution death group, an electrical injury group, a postmortem electrocution group, and a control group. Each group was then subdivided into 6–8 subgroups at 0 hr, 0.25 hr, 0.5 hr, 1 hr, 2 hr, 4 hr, 6 hr, and 8 hr. The details of the groupings are illustrated in Table 1. All animals were allowed to eat and drink freely at room temperature (25 ± 3°C), and all groups received normal feeding.

### 2.2. Ethics Statement

The rat care and use were conducted in strict accordance with the recommendations of the* Guide for the Care and Use of Laboratory Animals of the National Institutes of Health*. All protocols were approved by the Medical Animal Studies Committee of Xi'an Jiaotong University, China. SD rats were housed and bred in a temperature-controlled room, received a standard chow diet, and were maintained on a cycle of 12 h of light and 12 h of dark with the darkness beginning at 19:00. All of the procedures were performed with the animals under general anesthesia. The rats were sacrificed by cervical dislocation. All efforts were made to minimize suffering, and the only procedures performed on the dead animals were the collection of the blood, ventricles, and livers.

### 2.3. Animal Model Establishment

All procedures were performed with the animals under general anesthesia in all groups. The rats were intraperitoneally injected with urethane (5 mL/kg). After the effect of anesthesia was verified, the rats' left forelimbs and right hind limbs were wet with normal saline and wrapped with normal saline sponges.

The rats in the serological examination part were then shocked with 110 V alternating current for 30 s. After the electrical shock, the rats were subjected to cervical dislocation according to the scheduled timetable, and 4–6 mL blood was collected from the abdominal aorta, placed in a coagulation tube, and centrifuged. The serum was then collected for later detection. The rats in the blank subgroup were sacrificed by cervical dislocation, and the rats in the blank subgroup were subjected to the same operation but without the electrical shocks.

The rats in the pathological examination part were also shocked with 110 V of alternating current. The rats in the electrocution death group were shocked for 2 minutes. The rats in the electrical injury group were shocked for 30 s. The rats on the postmortem electrocution group were shocked for 30 s after they were sacrificed by cervical dislocation. The ventricles and livers of all groups were collected for pathological observation. The rats in the control group were sacrificed by cervical dislocation. The rats in the control group were given the same operation but without the electrical shocks.

### 2.4. Serological Detection

The serology section consisted of the detection of CK-MB, ALT, and AMS. The samples were taken from the blank subgroup and the other subgroups after administering the electric shocks at 0 hr, 0.25 hr, 0.5 hr, 1 hr, 2 hr, 3 hr, 4 hr, 5 hr, 6 hr, 7 hr, and 8 hr. The CK-MB, ALT, and AMS levels were determined with an automatic biochemistry analyzer (Hitachi biochemistry analyzer 7170A).

### 2.5. Histological Examination

The pathological examination consisted of hematoxylin-eosin staining (HE) detection and IHC staining for Bc1-2, Bax, and Hsp-60. The ventricle and liver (left lobe) were dissected and fixed with 4% paraformaldehyde. The samples were then embedded in paraffin, cut into 6 *μ*m slices, and deparaffinized. The antigens were repaired with citrate sodium and then incubated with anti-1 (anti-Bc1-2, Bax, or Hsp-60) overnight at 4°C. Anti-2 was used for a thermostatic effect for 15 min. The samples were colored with DAB, counterstained according to the directions, and mounted to observe and analyze the images with the IPP6.0 software.

### 2.6. Statistical Analysis

The SPSS 13.0 software (SPSS, US) was utilized to perform one-way ANOVAs and chi-squared tests. The measurement data are presented as the means ± the standard deviations ($\overset{-}{x}\pm s$). The SNK test was employed for the group comparisons. The significance level was set a *P* < 0.05.

## 3. Results

### 3.1. Serological Results

The data from the serological examination section are provided in Table 2.

The CK-MB of the rats significantly increased immediately after the shock to reach approximately 2.7-fold the amount in the blank subgroup at 0.5 hr (*P* < 0.05, one-way ANOVA). Before returning to the original level, another smaller peak occurred at 3 h after the electrical stimulation.

The ALT level rose slightly during the first hour to peak at 3 hr at approximately 1.5 times the level of the blank subgroup, and this difference was significant (*P* < 0.05, one-way ANOVA). The ALT level then decreased sharply and finally returned to the same level as the 0 hr subgroup at 8 h.

AMS rose significantly at approximately 0.5 hours after the operation and peaked at 3 h after the shock at a level of 1771.1 ± 95.8 U/L (mean ± SD). In the end, the AMS level decreased back to the level observed in the blank subgroup.

### 3.2. Protein Results

**Figure 1:**
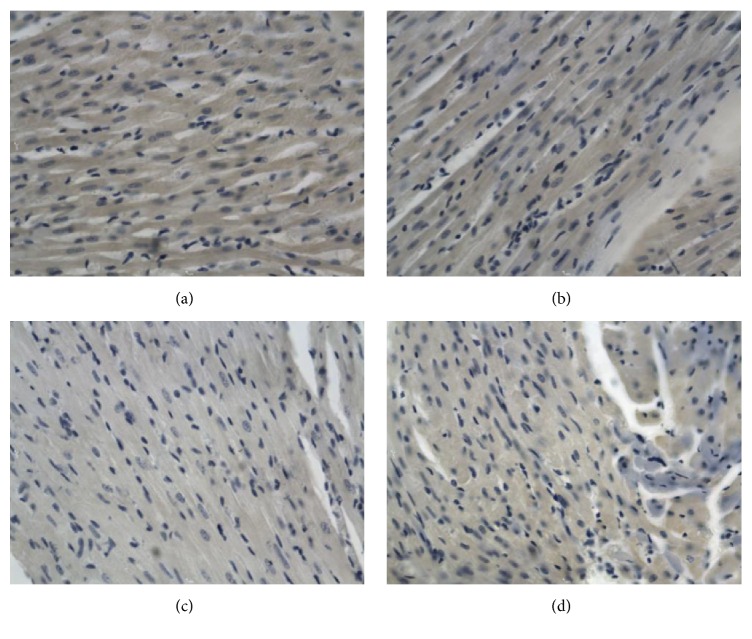
Bcl-2 in the ventricle after electric shock, DAB, 400x, 0.25 hr ((a) electrocution death group, (b) electrical injury group, (c) postmortem electrocution group, and (d) control group).

**Figure 2 fig2:**
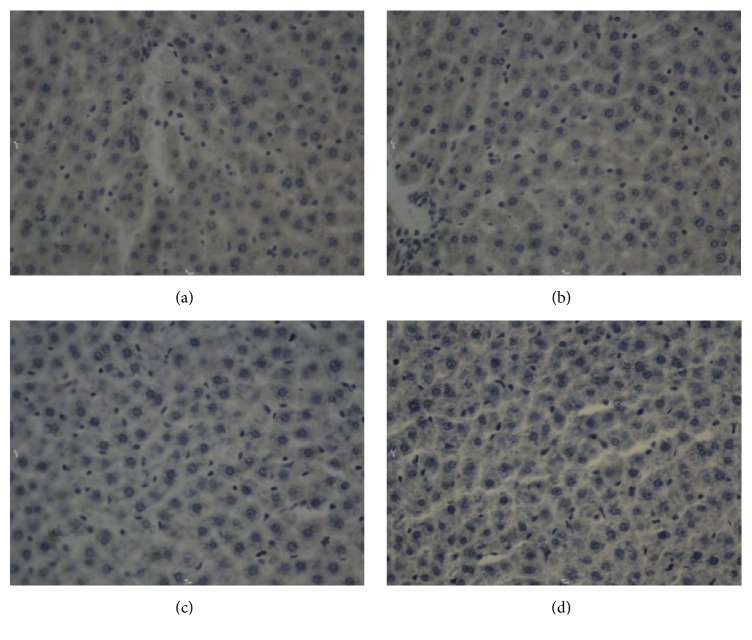
Bcl-2 in hepatocytes after electric shock, DAB, 400x, 0.25 hr ((a) electrocution death group, (b) electrical injury group, (c) postmortem electrocution group, and (d) control group).

**Figure 3 fig3:**
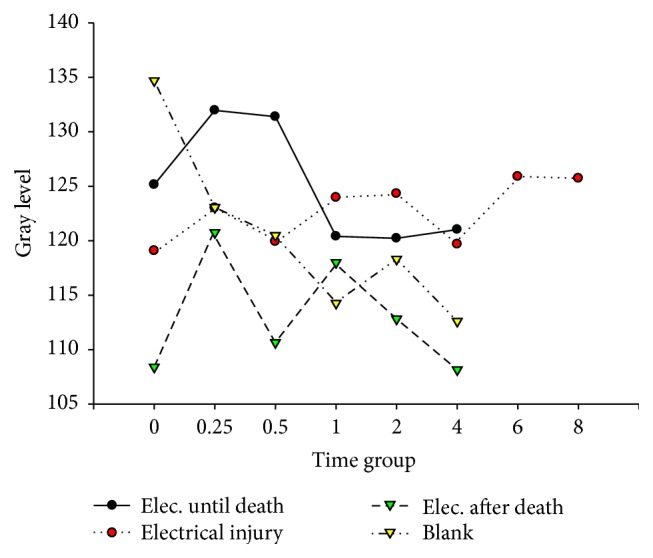
Bcl-2 in cardiac muscle after electric shock.

**Figure 4 fig4:**
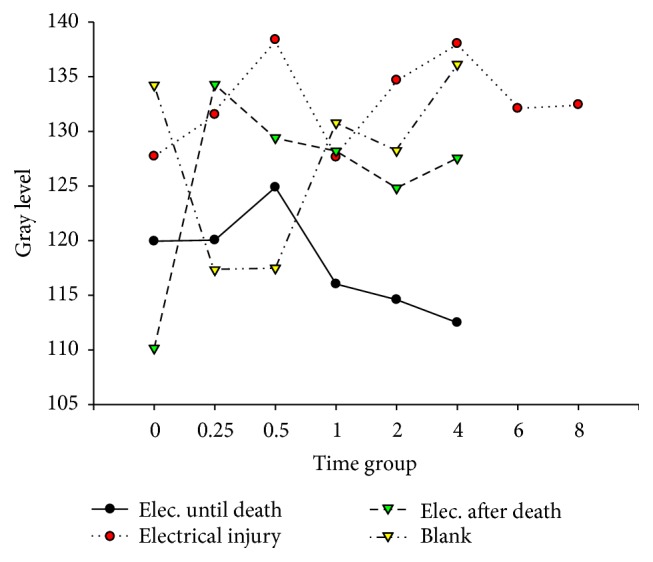
Bcl-2 in hepatocytes after electric shock.

**Figure 5 fig5:**
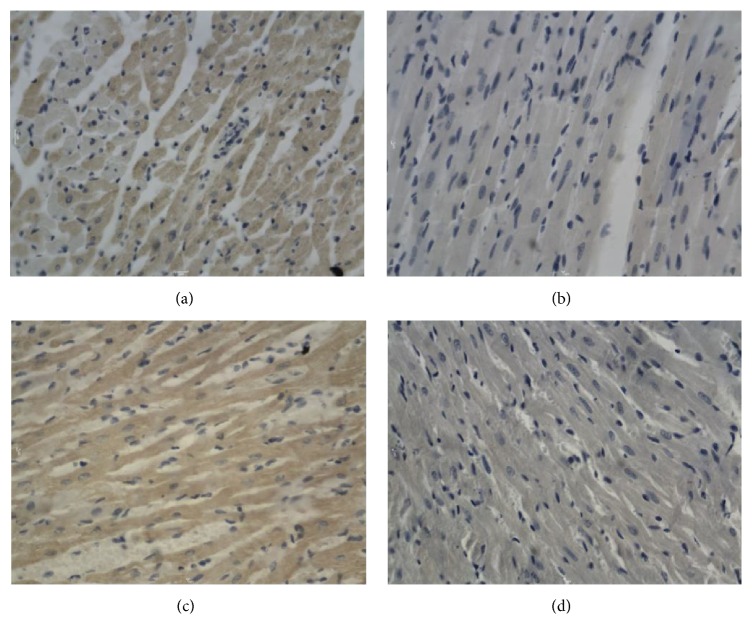
Bax in ventricle after electric shock, DAB, 400x, 0.25 hr ((a) electrocution death group, (b) electrical injury group, (c) postmortem electrocution group, and (d) control group).

**Figure 6 fig6:**
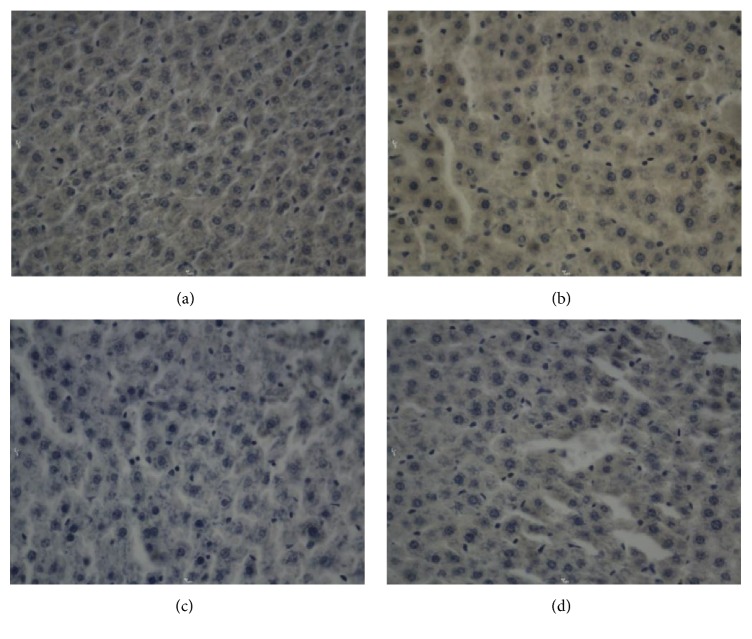
Bax in hepatocytes after electric shock, DAB, 400x, 0.25 hr ((a) electrocution death group, (b) electrical injury group, (c) postmortem electrocution group, and (d) control group).

**Figure 7 fig7:**
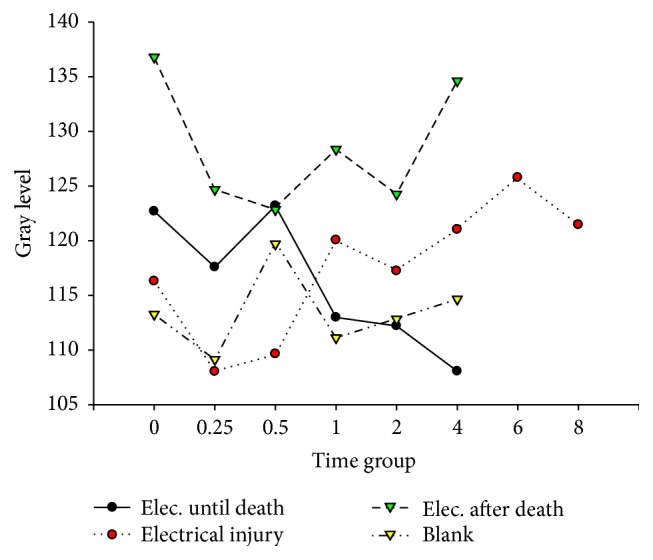
Bax in cardiac muscle after electric shock.

**Figure 8 fig8:**
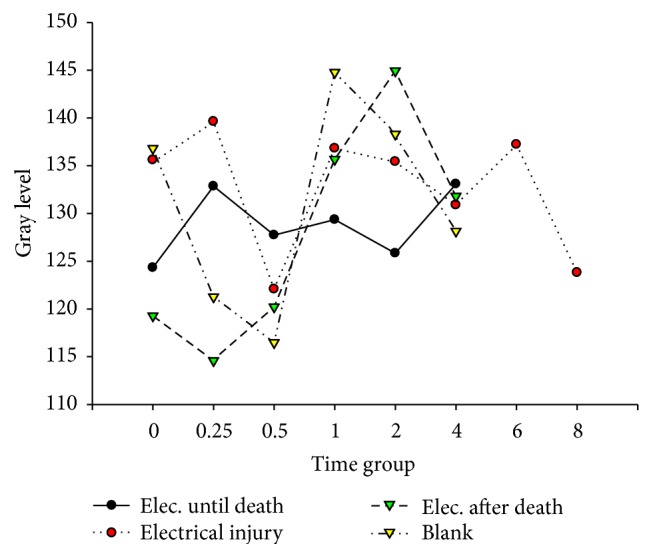
Bax in hepatocytes after electric shock.

**Figure 9 fig9:**
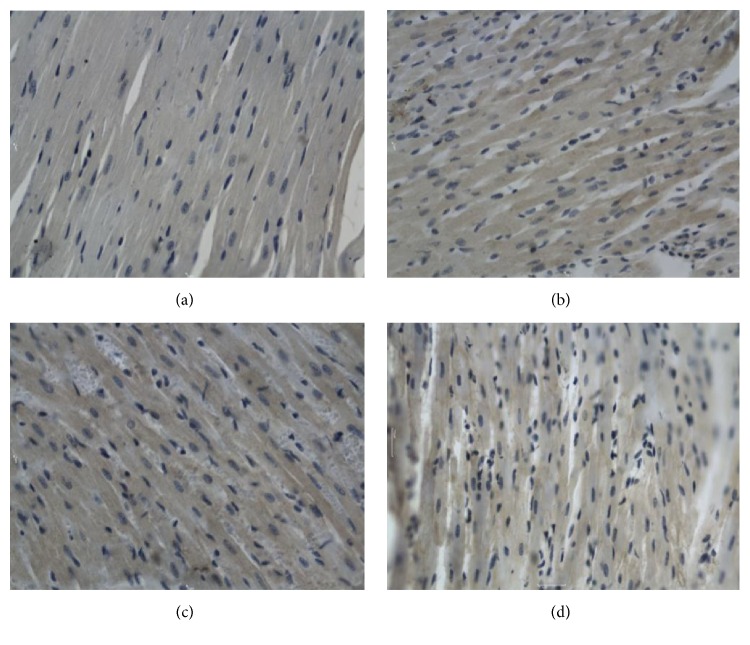
HSP in the ventricle after electric shock, DAB, 400x, 0.25 hr ((a) electrocution death group, (b) electrical injury group, (c) postmortem electrocution group, and (d) control group).

**Figure 10 fig10:**
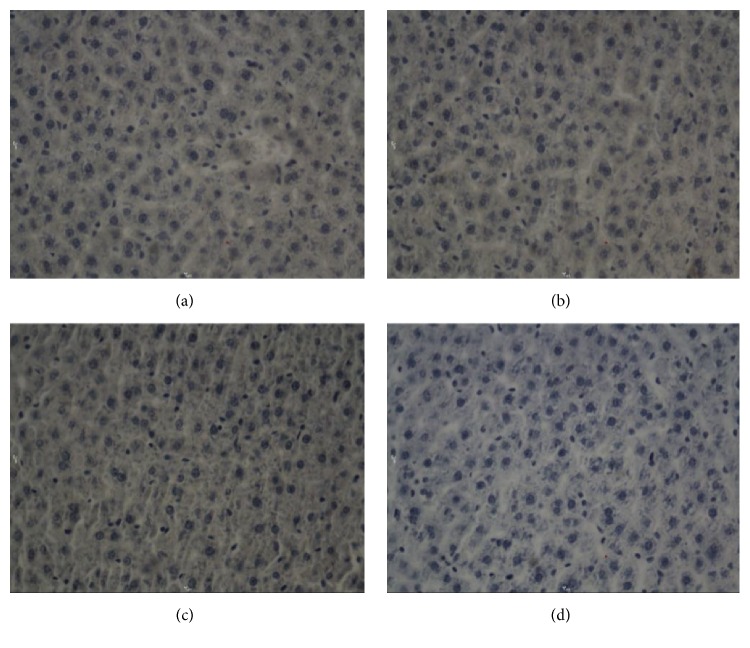
HSP in hepatocytes after electric shock, DAB, 400x, 0.25 hr ((a) electrocution death group, (b) electrical injury group, (c) postmortem electrocution group, and (d) control group).

**Figure 11 fig11:**
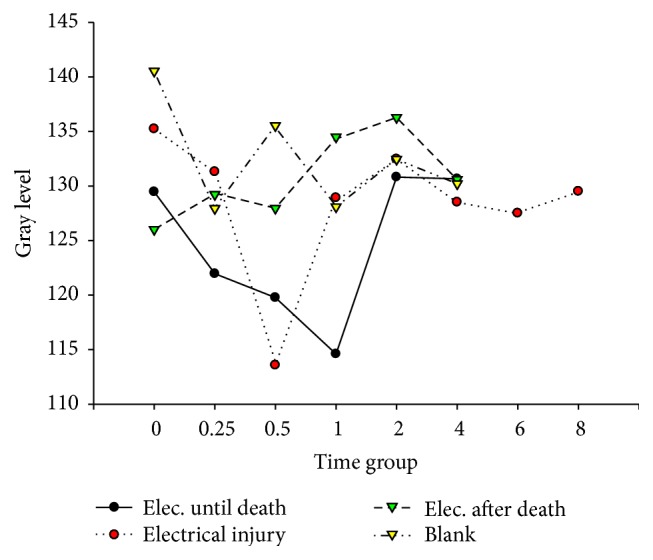
Hsp-60 in hepatocytes after electric shock.

**Figure 12:**
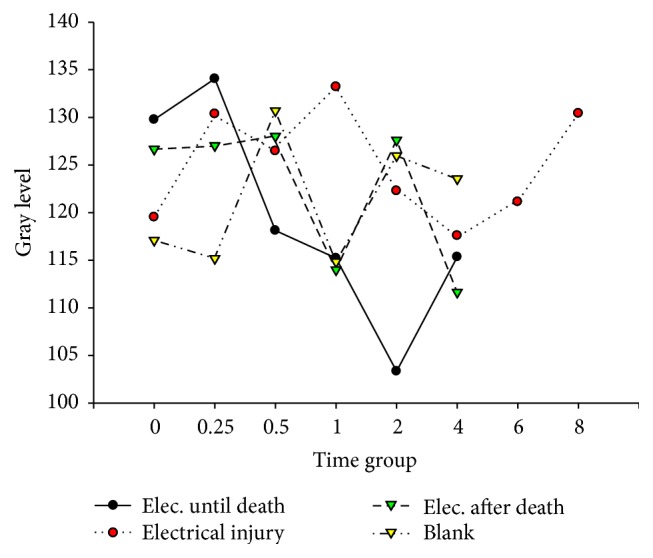
Hsp-60 in hepatocytes after electric shock.

All the data and results from pathological examination section are provided in Tables 3 [Table tab4] [Table tab5] [Table tab6] [Table tab7]–8 and Figures 1 [Fig fig2] [Fig fig3] [Fig fig4] [Fig fig5] [Fig fig6] [Fig fig7] [Fig fig8] [Fig fig9] [Fig fig10] [Fig fig11]–12. Our study revealed that, in the electrocution death group, the level of Bcl-2 in the myocardium reached a peak at 1 hr (*P* < 0.05, Figure 3, Table 3), and significant differences between the different time groups were noted. In contrast, the level of Bcl-2 at 0.5 hr after the electrical shock significantly increased in the hepatocytes (*P* < 0.05, Figure 4, Table 4). In the hepatocytes, the levels of Bax exhibited two obvious increases at 0.25 h (132.85, *P* < 0.05, Figure 8, Table 6) and 4 h (133.07, *P* < 0.05, Figure 8, Table 6). Moreover, the HSP-60 protein in the myocardium decreased significantly (114.60, *P* < 0.05, Figure 11, Table 7) in the 1 h group and reached the highest level (130.81, *P* < 0.05, Figure 11, Table 7) in the 2-hour group compared with the level in the hepatocytes, which exhibited a sharp reduction (103.34, *P* < 0.05, Figure 12, Table 8) in the 2 h group.

In the postmortem electrocution group, greater differences were observed. The BCL-2 levels in the myocardium and hepatocytes increased greatly in the 15 min group in which the level of BCL-2 in the hepatocytes was 134.28 (*P* < 0.05, compared with the electrocution group, the control group, and the previous time group, Figure 2). The Bax level in the myocardium exhibited two peaks at the beginning (136.78, *P* < 0.05, compared with the control group) and the end (134.672, *P* < 0.05, compared with the control group) of the experiment, whereas in the hepatocytes, the Bax level reached 144.92 (*P* < 0.05, compared with the control group, Figures 6 and 8, Table 6) 2 hours after the shock. Moreover, the HSP-60 level in the myocardium was found to begin at an intermediate level of 125 and reach the highest level of 136.28 in the 2 h group (*P* < 0.05 compared with the control group, Table 7). In contrast, in the hepatocytes, the HSP-60 level fluctuated during the experiment and exhibited a level of 127.60 in the two-hour group (*P* < 0.05 compared with the electrocution group, Table 8).

## 4. Discussion

Injury caused by electrical current is known as electrical injury and can easily and frequently occur due to the widespread use of electrical appliances [3]. Data reveal that 0.5% of deaths are related to electrical shocks, and of these deaths, 60%–70% are caused by low-voltage power supplies, including the occasional short circuit of the storage batteries of cars in America and China [7, 8].

A widely accepted mechanism of electrical injury is that the heat injury caused by an electrical current does direct damage to the tissues via electrical current and causes a secondary injury after the shock. Electrical arcs with high-tension power lines usually burn the tissues and increase the electrical resistance on the electrical spot. Therefore, it is difficult for electrical current to pass through vital organs, which results in a lower mortality rate but a higher injury rate [1, 4–6, 9]. However, if strong electrical current flows through heart, it may do a direct damage to the cardiac tissues and eventually cause deadly ventricular fibrillation and the quick death of the victims.

The diagnosis of electrical injury is a real problem. The presence of skin lesions and the pathological appearance of electrical injuries are usually nonspecific. Charred areas and blisters can be found in cases of both electrical burns and flame burns. Microscopically, epidermal nuclear elongation in a somewhat parallel fashion (nuclear streaming) and separation in the epidermis are also regarded as meaningful manifestations of electrical injury [10]. In special circumstances, the separation of epidermal cells also can be caused by evaporating tissue fluids depending on the increase in heat, and mild nuclear elongations may also be found in abrasions [11]. Crater-like areas of melted keratin, which are called electrical marks, are found more frequently with high- than low-voltage currents [12]. A number of low-voltage and short-time electrocution victims may exhibit only very subtle or no electrical marks. Consequently, the causes of injury or death are difficult to identify. An easy method for detecting electrical injury is needed.

Based on the aforementioned factual circumstances of low-voltage burns, we established this electrical model with the following characteristics: (1) we sought to investigate the electrical injuries at the systemic level, particularly in vital organs, rather than focusing only on the local skin and muscle injuries; (2) we attempted to identify some indexes that are normally used in some usual diseases that may also be useful for identifying electrical injuries; (3) an additional aim of this study was to identify a plausible explanation for the difference between ante- and postmortem electrical injuries. Therefore, we placed the electrodes on the rats' left forelimbs and right hind limbs and let the electrical current flow through the organs in the thorax and abdomen. The four previously mentioned groups were set up to simulate different conditions because electrical shocks might affect postmortem findings. Biao and his colleagues [13] found that the levels of CK and CK-MB in 32 patients who had experienced an electrical shock were significantly greater than those of normal people. PEG-3 in rat cardiac muscle is positively expressed in both electrocution and electrical injury groups [14]. Similarly, c-fos and caspase-8 are also more highly expressed in rat cardiac muscle after electrical injury [15]. Moreover, the levels of c-fos expression in antemortem electric injury groups are higher than those of postmortem injury groups, and c-fos oncogene protein expressions in electricity up to death groups are higher than those in antemortem electricity groups and postmortem electricity groups [16]. In the present study, we found that some indexes that are normally used in some usual diseases can be useful in identifying electrical injury. Additionally, we found that changes in some proteins in specific organs can also aid clinical and medicolegal experts in distinguishing electrical wounds in several complicated conditions.

### 4.1. Changes in CK-MB Levels after Electrical Injury

CK-MB is widely present in the cardiac muscle and is a marker of cardiac muscle injury that reflects the severity of the injury. In the present study, the myocardium was injured immediately after the electrical shock. According to Jia-ke and his colleagues [17], the levels of CK and CK-MB are also significantly elevated, particularly at 24 h after a high-voltage electrical injury. The level of CK-MB in the serum at 1 hour after electrical injury can be useful in clinical diagnoses, but the same change can be observed in other conditions, such as AMI and SAH [18, 19]. Moreover, electrical injury can damage the skeletal muscles, which also contain CK-MB. Therefore, we assume that Ck-MB in the skeletal muscles may be involved in the increase in CK-MB. Moreover, electrical injury can also occur in the brain. Injuries of the brainstem and medulla may negatively affect the load of the heart by eliciting a “secondary injury,” as demonstrated by the peak in the analysis.

### 4.2. Changes in ALT Levels after Electrical Injury

ALT is an important enzyme for human physiological activities. ALT catalyzes the transamination reaction between alanine and *α*-oxoglutarate in the metabolism of glucose and amino acids [20]. When hepatocytes are damaged, ALT is released into the blood, and the level of ALT increases in the serum. Therefore, ALT is one of the important markers for the quantitation of hepatocyte function.

In this study, we observed that the level of ALT in the serum changed regularly during the 8-hour experiment after electrical injury. The ALT level consistently increased after the electrical injury and peaked at 3 hours. This finding suggests that hepatocytes might experience extensive injuries shortly after electrical injury.

Generally, the release of enzymes indicates damage to the cell membrane. The injuries caused by the electrical currents in our research were in line with this supposition. In our study, prior to the ALT peak at 3 hours, the ALT levels fluctuated. This observation can be explained by the theory of electrical injury propounded by Lee [6], which implicate the involvement of cellar repair and absorption of the enzymes as well as the influence of membrane injury in electrical shock [21]. Wang et al. [22] conducted research regarding electrical injuries to multiple rat organs. However, it has not yet become explicit how the liver, pancreas, biliary system, and circulation system affect each other during electrical injury because this study was the first time that serological reactions were used in this field. Moreover, the difference between humans and rats cannot be overlooked.

### 4.3. Changes in AMS Levels after Electrical Injury

Amylase (AMS) can catalyze the hydrolysis of starch and glycogen. AMS is restored and secreted by the pancreas and the saliva and can also be found in the lungs, liver, thyroid, and fat. The detection of AMS in the serum is commonly used in the clinical diagnosis of acute pancreatitis [23]. Within 15 minutes, the level of AMS did not apparently increase, but 30 minutes later, the AMS level significantly increases to reach a peak at 3 hours after the experiment began. We believe that although the pancreas cells were injured by the electrical shock, the effects induced by the injury did not appear immediately. An electrical current can cause pancreatic heat injury [24]. Similarly, due to the blockade of the circulation system caused by fibrillation during electrical shock, the pancreas is unable to obtain enough oxygen, which results in membrane breaking and AMS leaking into the blood.

### 4.4. Changes in Bc1-2 Expression after Electric Shock

Bcl-2 is one of the most popular apoptosis-controlling genes and widely exists in the membranes of cells [25] where it participates in the processes of cell growth and apoptosis. The expression of Bcl-2 changes according to different conditions, such as electric shocks. Therefore, explaining electric injury at the molecular level is meaningful.

Deaths caused by electric shocks are always linked to ventricular fibrillation and cardiac arrest. The analysis of Bc1-2 expression reveals the effect of the protection protein after electrical shock. In this study, the myocardium was very likely injured after the electrical shock. The Bc1-2 level dropped instantly in a time-dependent manner following cell injury. In contrast, the expression in the electrocution death group exhibited an instant increase that lasted for 30 minutes. According to Lee [6], this effect may be caused by the disaggregation of the enzyme system and organelles. The activation and expression of Bc1-2 are seriously limited, especially in the myocardium, which is common in cancer cells [26]. Regarding the electrical injury group and the postmortem electrocution group, the electrical current may have been an unusual factor because the affecting time was short. In the electrocution death group, the electrical current might have increased Bc1-2, but this trend toward an increase could not last too long because of death. In forensic practice, victims who have died of electrical injury usually have been shocked for at least 10 minutes, which means that the increase in Bc1-2 expression could be detected. Thus, this index could help to determine whether victims have received an electrical injury. We also noticed the phenomenon that the expression of Bc1-2 was observable in the myocardium, which provides some ideas for further studies of cell differentiation and the occurrence of cancer.

In hepatocytes, the expression of Bc1-2 was greater than that in the myocardium. This change revealed that the hepatocytes could regenerate and had a high possibility of developing cancer [27]. This finding could probably explain the fact that the Bcl-2 level in the myocardium was so high that the cardiac cells were unlikely to be repaired after injury [28].

### 4.5. Expression of Bax after Electric Shock

The analysis of the changes in Bax, a proapoptotic protein that is expressed in the myocardium after electric shock, revealed that the levels of expression in the electrical injury groups were obviously greater than that in the control group. This finding indicated an unserious injury. However, the results were beyond our expectation; the Bax level did not decrease but rather increased. This effect might have been due to an idiosyncrasy of the myocardium. As a common fact, the myocardium is known as a “nonregenerative” tissue, especially regarding myocardial infarction. When the myocardium was injured by electric current, cell damage and apoptosis appeared, but the cell repair effect is not active as expected. All of these effects caused the Bax level to increase.

The level of Bax in the hepatocytes dropped to protect the cells, which parallels the degree of injury. However, due to the serious injury, a high level could not be sustained for a long time; thus, the level of Bax in the postmortem electrocution group lasted for a short time. After 15 minutes, the level of Bax in the electrocution death group increased, which indicated the loss of control of Bax synthesis. In contrast, in the electrocution death group, the injury was so serious that the cells may have lost control of expression. This phenomenon could explain the subsequent change and the increased level of Bax. We found that the electrocution death group was different from the others. This finding could help to distinguish short-term electric shock after death from death from electric shock.

### 4.6. Expression of Hsp-60 after Electric Shock

The damage from electrical shock includes heat injury, electroionization, and electrostimulation [5]. Heat injury and electrostimulation can lead the victim to a state of stress, which can activate the expression of stress proteins to help repairs. In our experiment, the myocardial level of Hsp-60 in the control group was higher than those in the electrocution death group and the postmortem electrocution group.

When the electric voltage is low, an electrical current easily induces respiratory muscle paralysis, respiratory failure, and asphyxia. In this experiment, a low voltage (110 V) was used; therefore, ventricular fibrillation, which might cause the myocardial consumption of oxygen, was likely to occur. Moreover, the oxygen supply was insufficient because of the respiratory muscle paralysis, which resulted in injuries of the myocardial mitochondria [29]. Consequently, the level of Hsp-60 began to increase. After death, the cells remained alive due to the consumption of the remaining oxygen. We thought that some of the vital organ activities still continued, which could explain the increase in the Hsp-60 level in the postmortem electrocution group. Four hours after the electric shock, the level of Hsp-60 in the electrocution death group was significantly lower than those of the other groups. Therefore, Hsp-60 may be helpful for evaluating electrical shock death and ensuring whether there was an electric shock after death.

The hepatocyte expression of Hsp-60 was the highest in the electrocution death group, which indicates that severe destruction had occurred. However, the level dropped at 30 minutes after the shock in the electrocution death group. We suspect that the cells could not continuously synthesize after the electrical injury and subsequent death, which resulted in a sharp level decline. Intriguingly, 2 hours later the level of Hsp-60 in the postmortem electrocution and control groups increased. According to Lee, this might be a “fake rise” that is due to the interruption of the physiological reaction of the protein [21].

## 5. Conclusion

By studying the different patterns of electrical injuries, we detected three serological index changes, studied the changes in the expressions of proteins, and summarized the pathological methods for determining the degree of electrical injuries to important organs. The work we have performed will not only help clinical doctors avoid misdiagnoses in complicated circumstances but also assist forensic experts in identifying electrical shock among various types of injury.

## Figures and Tables

**Table 1:** Grouping (*n* = 228).

Time subgroups	Serology examination section after electric shock	Pathological examination section			
		Electrocution death group	Electrical injury group	Postmortem electrocution group	Control group
Blank	6	—	—	—	—
0 hr	6	6	6	6	6
0.25 hr	6	6	6	6	6
0.5 hr	6	6	6	6	6
1 hr	6	6	6	6	6
2 hr	6	6	6	6	6
3 hr	6	—	—	—	—
4 hr	6	6	6	6	6
5 hr	6	—	—	—	—
6 hr	6	—	6	—	—
7 hr	6	—	—	—	—
8 hr	6	—	6	—	—

**Table 2:** Serology examination section after electric shock ($\overset{-}{x}\pm s$, *n* = 72).

Time subgroups	CK-MB (U/L)	ALT (U/L)	AMS (U/L)
Blank	1442.2 ± 202.8	42.3 ± 2.4	1341.3 ± 143.8
0 hr	1815.5 ± 496.8	49 ± 4	1587 ± 229
0.25 hr	2237.4 ± 376.5	45 ± 3.7	1506.3 ± 252.3
0.5 hr	3889.2 ± 1043.8^*∗*#^	46.7 ± 7.7	1623.5 ± 302.3^*∗*^
1 hr	2506 ± 585.8^*∗*#^	58 ± 11.2^*∗*^	1631.2 ± 100.1
2 hr	1698.4 ± 699.4	45 ± 9.7	1589 ± 130.3
3 hr	2213 ± 250.7	63 ± 9^*∗*^	1771.8 ± 95.8^*∗*^
4 hr	1713.5 ± 561	62 ± 8.7^*∗*^	1556.7 ± 128.3
5 hr	1921 ± 202.3	60.8 ± 16.8^*∗*^	1635.2 ± 177.2^*∗*^
6 hr	1143.5 ± 332.8	55.8 ± 8.2	1526.8 ± 171.5
7 hr	1292.7 ± 118	48.5 ± 10.8	1348.8 ± 124.8
8 hr	1206.3 ± 115.7	49.7 ± 9.8	1288.7 ± 111

^*∗*^ *P* < 0.05, compared with blank subgroup; ^#^ *P* < 0.05 compared with the previous subgroup.

**Table 3:** Bcl-2 in cardiac muscle after electric shock ($\overset{-}{x}\pm s$, *n* = 156).

Time subgroups	Electrocution death group	Electrical injury group	Postmortem electrocution group	Control group
0 hr	125.14 ± 12.21^#^	119.07 ± 19.87^#^	108.39 ± 19.84^*∗*#+^	134.69 ± 17.96
0.25 hr	131.96 ± 15.67	123.01 ± 16.56	120.74 ± 13.25^*∗*+^	123.05 ± 18.32^*∗*^
0.5 hr	131.38 ± 14.02^#^	119.91 ± 16.43	110.64 ± 21.25^#+^	120.47 ± 16.26
1 hr	120.39 ± 13.66^*∗*^	123.96 ± 13.73^#^	117.99 ± 16.14	114.25 ± 33.98
2 hr	120.21 ± 14.85	124.31 ± 14.46	112.80 ± 18.93	118.30 ± 22.61
4 hr	121.01 ± 14.87	119.67 ± 17.85	108.14 ± 18.06^*∗*+^	112.59 ± 24.38
6 hr	—	125.88 ± 13.77	—	—
8 hr	—	125.72 ± 10.70	—	—

^*∗*^ *P* < 0.05, compared with the previous subgroup; ^#^ *P* < 0.05 compared with the control group; ^+^ *P* < 0.05 compared with electrocution death group.

**Table 4 tab4:** Bcl-2 in hepatocyte after electric shock ($\overset{-}{x}\pm s$, *n* = 156).

Time subgroups	Electrocution death group	Electrical injury group	Postmortem electrocution group	Control group
0 hr	119.94 ± 5.60^#^	127.72 ± 4.17	110.15 ± 9.42^#+^	134.21 ± 13.10
0.25 hr	120.04 ± 3.73	131.54 ± 5.56^#^	134.28 ± 3.59^*∗*#+^	117.32 ± 7.27^*∗*^
0.5 hr	124.88 ± 3.48	138.38 ± 4.43^#^	129.41 ± 5.85^#^	117.47 ± 3.54
1 hr	116.03 ± 4.70^*∗*#^	127.63 ± 12.21^*∗*^	128.19 ± 3.43^+^	130.76 ± 2.07^*∗*^
2 hr	114.59 ± 4.46^#^	134.67 ± 11.78	124.80 ± 7.90^+^	128.23 ± 10.79
4 hr	112.49 ± 5.37^#^	138.02 ± 1.74	127.55 ± 8.28^#+^	136.12 ± 2.11^*∗*^
6 hr		132.10 ± 3.44		
8 hr		132.43 ± 7.46		

^*∗*^ *P* < 0.05, compared with the previous subgroup; ^#^ *P* < 0.05 compared with the control group; ^+^ *P* < 0.05 compared with electrocution death group.

**Table 5 tab5:** Bax in cardiac muscle after electric shock ($\overset{-}{x}\pm s$, *n* = 156).

Time subgroups	Electrocution death group	Electrical injury group	Postmortem electrocution group	Control group
0 hr	122.69 ± 10.47^#^	116.30 ± 25.10	136.78 ± 26.22^+^	113.26 ± 24.78
0.25 hr	117.58 ± 15.88^#^	108.03 ± 22.67	124.68 ± 23.45	109.14 ± 26.97
0.5 hr	123.19 ± 15.29	109.64 ± 27.43	122.84 ± 15.76	119.71 ± 26.26
1 hr	112.98 ± 20.85^#^	120.05 ± 36.13	128.35 ± 25.19^+^	111.12 ± 19.46
2 hr	112.20 ± 8.55	117.23 ± 38.51	124.27 ± 24.36	112.82 ± 26.38
4 hr	108.06 ± 9.85^#^	121.02 ± 29.65	134.62 ± 23.00	114.65 ± 30.94
6 hr		125.77 ± 22.56		
8 hr		121.46 ± 21.36		

^#^ *P* < 0.05 compared with the control group; ^+^ *P* < 0.05 compared with electrocution death group.

**Table 6 tab6:** Bax in hepatocyte after electric shock ($\overset{-}{x}\pm s$, *n* = 156).

Time subgroups	Electrocution death group	Electrical injury group	Postmortem electrocution group	Control group
0 hr	124.32 ± 1.56^#^	135.58 ± 4.02	119.27 ± 7.81^#^	136.81 ± 4.93
0.25 hr	132.85 ± 6.52^*∗*#^	139.62 ± 0.64^#^	114.58 ± 4.94^#+^	121.26 ± 2.11^*∗*^
0.5 hr	127.73 ± 4.89^#^	122.09 ± 7.00^*∗*#^	120.21 ± 3.08^*∗*+^	116.47 ± 1.07
1 hr	129.35 ± 5.07^#^	136.81 ± 2.13^*∗*#^	135.66 ± 3.84^*∗*#+^	144.75 ± 2.71^*∗*^
2 hr	125.83 ± 4.61^#^	135.42 ± 2.28	144.92 ± 1.01^*∗*#+^	138.30 ± 2.09^*∗*^
4 hr	133.07 ± 3.27^*∗*^	130.88 ± 3.82	131.80 ± 6.21^*∗*^	128.14 ± 2.08^*∗*^
6 hr		137.21 ± 2.93^*∗*^		
8 hr		123.81 ± 6.07^*∗*^		

^*∗*^ *P* < 0.05, compared with the previous subgroup; ^#^ *P* < 0.05 compared with the control group; ^+^ *P* < 0.05 compared with electrocution death group.

**Table 7 tab7:** Hsp-60 in cardiac muscle after electric shock ($\overset{-}{x}\pm s$, *n* = 156).

Time subgroups	Electrocution death group	Electrical injury group	Postmortem electrocution group	Control group
0 hr	129.47 ± 2.09^#^	135.23 ± 11.02	126.01 ± 2.29^#^	140.52 ± 4.49
0.25 hr	121.96 ± 11.76^#^	131.308 ± 9.27	129.22 ± 7.40^+^	127.94 ± 9.00^*∗*^
0.5 hr	119.76 ± 10.54^*∗*#^	113.56 ± 10.28^*∗*#^	127.96 ± 4.63^#+^	135.53 ± 5.35^*∗*^
1 hr	114.60 ± 13.06^*∗*#^	128.91 ± 4.12^*∗*^	134.50 ± 3.76^*∗*#+^	128.08 ± 5.06^*∗*^
2 hr	130.81 ± 4.32^*∗*^	132.46 ± 14.58	136.28 ± 4.51^+^	132.45 ± 6.76
4 hr	130.65 ± 3.97	128.51 ± 4.53	130.59 ± 8.18^*∗*^	130.20 ± 2.31
6 hr		127.50 ± 3.43		
8 hr		129.50 ± 2.39		

^*∗*^ *P* < 0.05, compared with the previous subgroup; ^#^ *P* < 0.05 compared with the control group; ^+^ *P* < 0.05 compared with electrocution death group.

**Table 8:** Hsp-60 in hepatocyte after electric shock ($\overset{-}{x}\pm s$, *n* = 156).

Time subgroups	Electrocution death group	Electrical injury group	Postmortem electrocution group	Control group
0 hr	129.76 ± 1.86^#^	119.52 ± 1.18	126.66 ± 7.79^#^	117.09 ± 5.31
0.25 hr	134.05 ± 5.23^#^	130.35 ± 1.01^#^	127.03 ± 1.49^#+^	115.18 ± 3.18
0.5 hr	118.11 ± 7.29^*∗*#^	126.46 ± 1.30^*∗*^	128.00 ± 4.08^+^	130.69 ± 0.69^*∗*^
1 hr	115.21 ± 2.04	133.21 ± 1.38^*∗*#^	113.99 ± 8.40^*∗*^	114.83 ± 4.28^*∗*^
2 hr	103.34 ± 2.25^*∗*#^	122.28 ± 6.10^*∗*^	127.60 ± 4.32^*∗*+^	127.01 ± 6.01^*∗*^
4 hr	115.34 ± 1.99^*∗*#^	117.58 ± 6.79	111.63 ± 7.93^*∗*#^	123.55 ± 3.05
6 hr		121.12 ± 8.06		
8 hr		130.41 ± 6.08^*∗*^		

^*∗*^ *P* < 0.05, compared with the previous subgroup; ^#^ *P* < 0.05 compared with the control group; ^+^ *P* < 0.05 compared with electrocution death group.
